# Novel application of hydrophobin in medical science: a drug carrier for improving serum stability

**DOI:** 10.1038/srep26461

**Published:** 2016-05-23

**Authors:** Liqiang Zhao, Haijin Xu, Ying Li, Dongmin Song, Xiangxiang Wang, Mingqiang Qiao, Min Gong

**Affiliations:** 1 The Key Laboratory of Molecular Microbiology and Technology, Ministry of Education, Nankai University, Tianjin, China; 2 Tianjin Neurological Institute, Tianjin Medical University General hospital, Tianjin, China; 3 Tianjin Institute of Pharmaceutical Research, Tianjin, China; 4 Department of Pharmacy, Tianjin Medical University, Tianjin, China; 5 University of Oxford, Oxford, UK.

## Abstract

Multiple physiological properties of glucagon-like peptide-1 (GLP-1) ensure that it is a promising drug candidate for the treatment of type 2 diabetes. However, the *in vivo* half-life of GLP-1 is short because of rapid degradation by dipeptidyl peptidase-IV (DPP-IV) and renal clearance. The poor serum stability of GLP-1 has significantly limited its clinical utility, although many studies are focused on extending the serum stability of this molecule. Hydrophobin, a self-assembling protein, was first applied as drug carrier to stabilize GLP-1 against protease degradation by forming a cavity. The glucose tolerance test clarified that the complex retained blood glucose clearance activity for 72 hours suggesting that this complex might be utilized as a drug candidate administered every 2–3 days. Additionally, it was found that the mutagenesis of hydrophobin preferred a unique pH condition for self-assembly. These findings suggested that hydrophobin might be a powerful tool as a drug carrier or a pH sensitive drug-release compound. The novel pharmaceutical applications of hydrophobin might result in future widespread interest in hydrophobin.

## Introduction

### Physiological characterizations of Glucagon Like Peptide-1 (GLP-1)

GLP-1, which was discovered in 1990, is a gut hormone released from intestinal L cells following oral glucose administration^1^. The function of GLP-1 is to stimulate the secretion of insulin, which balances abnormal blood glucose levels^2^,^3^,^4^,^5^,^6^. Compared with the administration of insulin directly, GLP-1 is an intelligent approach to achieve blood glucose control in a blood glucose level-dependent manner in which GLP-1 stimulates the secretion of insulin based on increased glucose levels. In healthy conditions, the function of GLP-1 is halted to avoid the risk of hypoglycemia, which is a main side-effect of insulin for type 2 diabetic patients. GLP-1 has been shown to regulate blood glucose concentrations by mechanisms including enhanced insulin synthesis/secretion, suppressed glucagon secretion, slowed gastric emptying, and enhanced satiety^7^. GLP-1 was also capable of inhibiting the apoptosis of β-cells, suggesting that GLP-1 might recover the β-cell functions that are injured in the patient’s condition^8^. The distinct clinical utility of GLP-1 makes it a potent therapeutic strategy for type 2 diabetes mellitus (T2DM).

Recent studies reported the identification of GLP-1 receptors in the heart, kidneys, and blood vessels, leading to investigations into the role of GLP-1 in cardiovascular function or cardiovascular disease (CVD)^9^,^10^,^11^. Preclinical studies provided evidence that GLP-1 favorably affected endothelial function, sodium excretion, recovery from ischemic injury, and myocardial function in animals^12^,^13^,^14^,^15^. Preliminary data also indicated that GLP-1 reduced markers of CVD risk, such as C-reactive protein and plasminogen activator inhibitor-1^16^,^17^,^18^.

However, the poor serum stability of GLP-1 (3–5 min) has significantly limited its clinical utility because of the rapid degradation catalyzed by the enzyme dipeptidyl peptidase IV (DPP-IV)^19^. The extremely poor serum stability renders the therapeutic administration of GLP-1 impractical; therefore, many efforts have focused on altering the pharmacokinetic properties of GLP-1 by developing a series of complexes and analogs, such as exenatide, liraglutide and albiglutide^20^. The active region in the GLP-1 molecule is also the degradation domain (the HA-fragment at the N-terminus of GLP-1), which obstructs modifications on the GLP-1 molecule^21^. Scientists at Eli Lilly found that exendin-4, a peptide produced in the salivary glands of the Gila monster (*Heloderma suspectum*), possesses similar glucose regulatory function to the human GLP-1 peptide. Following the FDA approval of exendin-4, liraglutide and albiglutide, which are long-acting GLP-1 analogs using palmitic acid conjugation and albumin fusion, respectively, were approved. Many other strategies have also been employed to achieve long-acting activity of GLP-1, including dimerization, intra-molecular conjugation, and additional variant positive charged amino acids on the N terminus^21^,^22^,^23^,^24^,^25^.

In this study, we attempted to extend the serum stability of GLP-1 by utilizing hydrophobin, a protein with self-assembling activity. Hydrophobin might wrap the GLP-1 molecules inside the self-assembled cavity and protect against the degradation of GLP-1 by proteases.

### Hydrophobin protein as a drug carrier

Hydrophobin, a crucial protein in fungal growth and development, enables the escape of hyphae from the aqueous environment into the air by making amphipathic structures on the hyphal surface^26^,^27^. The most important feature of hydrophobin is the ability to form an amphipathic membrane 10 nm thick, which could reverse the properties of the interface that is being coated. In fungi, the hydrophobic surface with a hydrophobin coating could facilitate the attachment of hyphae to hydrophobic surfaces, aerial growth of the hypha, dispersal of aerial spores, and proper gas exchange in fungal air channels^27^,^28^,^29^. Like hydrogel and amphipathic peptide, the self-assembly of hydrophobins is interesting for many applications, including personal care and emulsions, separation technologies, and biosensors. The unique property of hydrophobin is thought to make this protein a powerful potential drug carrier^30^,^31^,^32^,^33^,^34^,^35^. In Valo’s group, a nanoparticle was recently prepared with a coating of a hydrophobin protein, and a lipophilic drug (beclomethasone dipropionate) was wrapped into the cavity formed by the hydrophobin protein. Stabilization data indicated that this complex was stable for at least 5 h in suspension and for longer after freeze-drying^31^,^34^.

In this study, the hydrophobin protein was utilized to form a stable complex with GLP-1 to investigate if the cavity formed by the hydrophobin protein is capable of wrapping the GLP-1 molecules inside and extending the half-life of GLP-1. The stabilization assays were performed *in vitro* and *in vivo* to determine the increase in the half-life of GLP-1, and physiological properties, including insulin stimulation, cAMP accumulation and blood glucose regulation, were investigated.

## Materials and Methods

### Materials

DPP IV enzyme (0.1 mg/ml; ~95% purity) and human serum albumin were purchased from Sigma-Aldrich. Human GLP-1 (7-37) ELISA kits were purchased from Millipore, Inc., and cAMP kits were purchased from CisBio Bioassays. The rat INS-1 cell line was obtained from Ying Li of Tianjin Medical University General Hospital. A rat insulin detection kit was purchased from Phoenix Technology, Inc. A one–touch blood glucose meter and filters were purchased from Abbott. Other chemicals were purchased from Sigma unless otherwise specified.

### Animals

All studies were carried out with permits from the Animal Experiments Inspectorate, China. Animal protocols in current study were approved by the Administrative Panel on Laboratory Animal Care in Tianjin, China and the protocols followed the guideline in China and AAALAC protocol (http://www.aaalac.org/) for the Care and Use of Animals in Research.

Male ZDF (fa/fa) rats, lean male ZDF rats, and male Wistar rats were purchased from Shanghai Laboratory Animal Co. (SLAC), China Academy of Sciences (Shanghai, China). Rats were maintained in controlled temperature (21–23 °C) and light (on at 0800 h, off at 1900 h) with *ad libitum* access to food (RM1 diet, SLAC, Shanghai, China) and water except 4 h before glucose or peptides administrations.

### Protein expression and purification

The *P. pastoris* GS115 His^−^ cells containing transformed pPIC 9-*hgfI* (wild-type hydrophobin protein) or pPIC-*hgfI E24K* (mutated at the 24^th^ residue) were preserved in Professor Qiao’s laboratory^36^,^37^. The mutant was constructed based on the improvement of self-assembling activity. The proteins were expressed and purified as described previously^37^. The purified proteins were identified by western blotting using an anti-HGFI polyclonal antibody.

### Peptide Synthesis

GLP-1 peptide was ordered from the Peptide Center of Wuxi AppTec Company (Shanghai, China). Peptide was synthesized using solid-phase peptide synthesis (Liberty 1, CEM). The synthesized peptide was purified by a Surveyor HPLC system through a Waters Prep Nova-Pak HR C18 silica gel column (5 × 30 cm, 6 μm particle size, 6 nm pore size); the UV detection wavelength was set at 220 nm. The column was eluted at a flow rate of 0.5 ml/min in H_2_O containing 40% acetonitrile and 0.1% trifluoroacetic acid (TFA). The freeze-dried peptides were weighed and dissolved in saline to make 1 mg/ml stock solutions for further analyses.

### Analysis of the mixtures of GLP-1 and the hydrophobin proteins by HPLC

The wild-type hydrophobin and mutant were mixed with GLP-1 in phosphate buffer, pH 3.0, 7.0 and 10.0. The molecular ratios in mixture were remained at 1:1, 1:5, 1:10 and 1:20, respectively. The mixtures were sonicated for 30 seconds and then cool down slowly in 4 °C overnight. Each mixture (10 μl) was analyzed by a Surveyor HPLC system through a C18 analytical column (Thermal Scientific, USA). The column was eluted at a flow rate of 0.5 ml/min in a gradient mode with a mixture of mobile phase A (H_2_O + 25% acetonitrile) and mobile phase B (100% acetonitrile). Mobile phase A was eluted for 10 min, and thereafter mobile phase B was increased from 15% to 70% over a 40 min period. HPLC analyses were performed at ambient temperature and the UV detection wavelength was set at 210 nm. Ten microlitre aliquots of GLP-1 solution (10 μM) and hydrophobin (0.1 μM) were injected into the C18 column of the HPLC as the controls.

### Stabilization study of the hydrophobin and GLP-1 complex

The stabilities of wild-type hydrophobin/GLP-1 and hydrophobin E24K/GLP-1 complexes were investigated to determine whether the complex protects GLP-1 against protease degradation. Two complexes, hydrophobin/GLP-1 (pH 7.0, molar ratio of 1:10) and hydrophobin E24K/GLP (pH 4.0, molar ratio of 1:10) were administered subcutaneously to male Wistar rats (500 μg GLP-1/kg body weight, n = 6 per group), and blood samples from the rats were directly drawn into P700*, K_2_EDTA tubes (BD Biosciences, Franklin Lakes, NJ) by venipuncture at 0.5, 1, 4, 12, 24, 48, 72 and 96 h. Serum samples (200 μl) were immediately analyzed using a native GLP-1 (7-37) ELISA kit after being centrifuged for 20 min at 13,000 rpm. The amount of free GLP-1 measured in this assay was considered an indicator of the serum serum stability of the complex.

### Glucoregulatory assay

To determine whether complexes containing GLP-1 possessed glucose regulation activity, single dose glucoregulatory assays were performed. In this assay, the complexes were administered once into Wistar rats, and the blood glucose levels were monitored over 96 h. It was presumed that the apparent half-lives of the complex could be obtained by this single-dose glucose tolerance test, which would be beneficial for the determination of the administration frequency in further long-term glucose tolerance tests.

Each complex (100 μg GLP-1/kg body weight) was subcutaneously injected into fasting male Wistar rats (n = 7 per group) 30 min prior to glucose administration. GLP-1 and saline were injected into the control animals. Rats were given 2 g glucose/kg body weight via intraperitoneal injections. Blood was drawn from the tail vein, and glucose levels were measured using a glucometer 30 min after glucose administration. Chronic glucose injections (2 g/kg body weight) were administered 30 min prior to each blood glucose measurement time point during the 96-h experimental period. In this assay, the hydrophobin/GLP-1 complex prepared at pH 4.0 and the hydrophobin E24K/GLP-1 complex prepared in pH 7.0 were also utilized for ascertaining the incomplete assembly shown in HPLC assays.

After observations of the prolonged glucose clearance activity of the complex, the dosage-effect relationship was investigated for this complex in 48-h experiment periods at dosages of 10, 250, and 1250 μg GLP-1/kg body weight, respectively.

### Insulin stimulation assay

The insulin secretion assay was performed by injecting the complex (100 μg GLP-1/kg body weight) into male Wistar rats (n = 3 per group). Wild-type GLP-1 (100 μg/kg body weight) and saline were injected subcutaneously as controls. Glucose (10 g/kg body weight, a standard dose for oral glucose administration) was orally administered 30 min after injections of the complexes. Blood samples were collected by tail vein incision 5, 10, 20, 45, 90, 120, and 150 min after glucose administration. Blood samples were assayed for insulin levels using a rat insulin RIA kit.

### cAMP accumulation measurement

For the cAMP assay, INS-1 cells (1.0 × 10^5^ cells) were seeded and plated in each well of a 96-well opaque white plate. After 24 h, the media was replaced with RPMI 1640 medium containing 500 μM 3-isobutyl-1-methylxanthine (IBMX, an inhibitor of cAMP phosphodiesterase). Subsequently, 10 μg of GLP-1 or complex was added. The assay plate was treated with 2.5 μl/well cAMP and an equal volume of anti-cAMP conjugate (Cisbio) after a 1 h incubation. The homogenous time-resolved fluorescence (HTRF) signal was read on a SpectraMax M5 (Molecular Device) microplate reader. The ratio of absorbance at 665 nm and 620 nm (×10,000) was calculated and plotted.

### Potent anti-diabetic activity of complex

Because of the long-acting properties demonstrated by the complexes, the long-term glucose tolerance in ZDF rats was investigated to determine the anti-diabetic activity of the complexes. Male ZDF rats (n = 11 per group) were treated with complex (250 μg/kg/3 days) for the entire experimental period (46 days). The control groups were injected with wild-type GLP-1 (250 μg/3 days). HbA_1c_ levels were assessed using a DCA 200 analyzer (Bayer Diagnostics), and glucose levels were monitored during the experimental period.

### Statistical analyses

Student’s *t*-test was used to analyze the data. Unless otherwise stated, the results are reported as the mean ± standard error. P values less than 0.05 were considered significant.

## Results

### Formation of the hydrophobin/GLP-1 complex in a self-assembling manner

**Figure 1:**
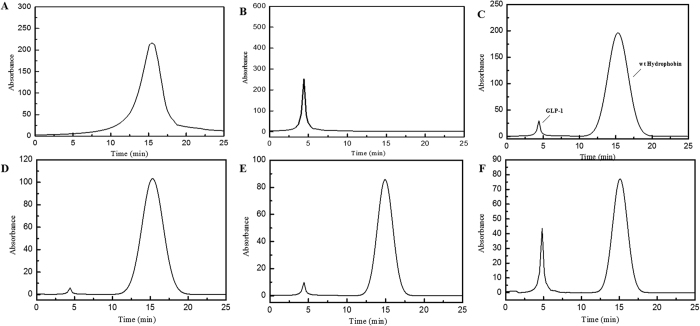
HPLC Comparison of GLP-1, wild-type hydrophobin and hydrophobin/GLP-1 mixed by different ratios. Panel A. HPLC spectrum of hydrophobin. Panel B. HPLC spectrum of GLP-1. Panel C. HPLC spectrum of the mixture of hydrophobin and GLP-1 at the ratio of 1:1. Panel D. HPLC spectrum of the mixture of hydrophobin and GLP-1 at the ratio of 1:5. Panel E. HPLC spectrum of the mixture of hydrophobin and GLP-1 at the ratio of 1:10. Panel F. HPLC spectrum of the mixture of hydrophobin and GLP-1 at the ratio of 1:20 Conditions: Samples were injected into C18 column of HPLC at 0.5 ml/min in solution containing 15% acetonitrile. UV detector was set at 220 nm. LEGEND: The retention times of GLP-1 and hydrophobin were 4.5 min (Fig. 1A) and 12.5 min (Fig. 1B). The peak of GLP-1 was significantly decreased at mixing ratio of 1:1 (Fig. 1C), 1:5 (Fig. 1D) and 1:10 (Fig. 1E), however GLP-1 re-observable upon the ration at 1:20 (Fig. 1F) suggesting the presence of exceeding GLP-1 molecules.

The identification of complex formation was carried out by RP-HPLC, and the results suggest that pH conditions strongly affect the assembly of the protein and peptide. For the wild-type hydrophobin/GLP-1 complex, the peak of GLP-1 completely disappeared at ratios of 1:5 and 1:10 (Fig. 1D,E), and then extra GLP-1 was observable at a retention time of 4.5 min when the molar ratio increased to 1:20 (Fig. 1F). However, the hydrophobin/GLP-1 complexes prepared at pH 7.0 and pH 10.0 showed different features; the peak of GLP-1 remained observable even at a ratio of 1:10 (data not shown). For the mutant in which the pI shifts to 5.8, the complex prepared at pH 7.0 showed assembling features on the HPLC spectrum. Similarly, the mutated hydrophobin lost the self-assembly property under neutral and basic conditions.

### Stabilization of the hydrophobin/GLP-1 complexes in rats

**Figure 2:**
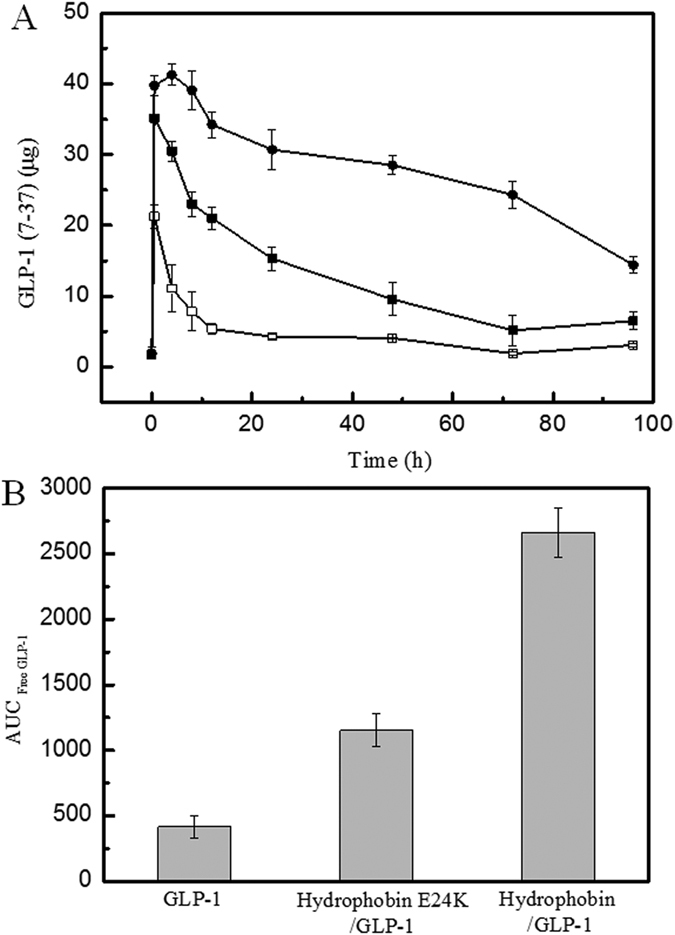
Stabilization studies of the complexes in rats. Panel A. A pharmacokinetic study of proteins and GLP-1 complex in Wistar rats. LEGEND: The results indicate that the wild-type hydrophobin/GLP-1 (●) and hydrophobin E24K/GLP-1 complex (■) were still detectable at 48 h, whereas native GLP-1 (□) was rapidly degraded within 25 min, showing that these complexes have prolonged stabilities. Panel B. AUC _GLP-1 (7-37)_ of the circulating GLP-1 concentration. Legend: The calculated AUC _GLP-1 (7-37)_ shows that the amount of complexes were significantly increased compared to native GLP-1, P < 0.01. Conditions: A human GLP-1 (7-37) ELISA kit was used to quantitatively determine the half-life of GLP-1 in rats, according to the manufacturer’s instructions.

To investigate if the shell formed by hydrophobin protected GLP-1 against protease degradation, serum stability assay was performed to determine the circulating concentration of free GLP-1 in rats using an ELISA kit. The data in Fig. 2A showed that after rats are given GLP-1, the concentration of GLP-1 increased significantly and was undetectable 4 h after administration because of rapid proteolysis. The rats treated with the hydrophobin/GLP-1 (pH 3.0) or hydrophobin E24K/GLP-1 complexes (pH 7.0) were characterized by similar features; the concentration of GLP-1 reached a plateau at 8 h. However, hydrophobin/GLP-1 exhibited better serum stability compared with the mutant. The GLP-1 leaking from the hydrophobin complex was still detectable at 96 h, but not from the hydrophobin E24K mutant complex (Fig. 2A).

The calculated AUC _free GLP-1_ within 96 h indicated that the amount of GLP-1 inside the hydrophobin/GLP-1 complex and the hydrophobin E24K/GLP-1 complex was increased 6.3 and 3-fold compared to that of GLP-1 alone (Fig. 2B). These data demonstrate that wild-type hydrophobin and the E24K mutant are able to significantly extend the half-life of GLP-1 in Wistar rats.

### Effects of complexes on insulin secretion and cAMP accumulation

**Figure 3:**
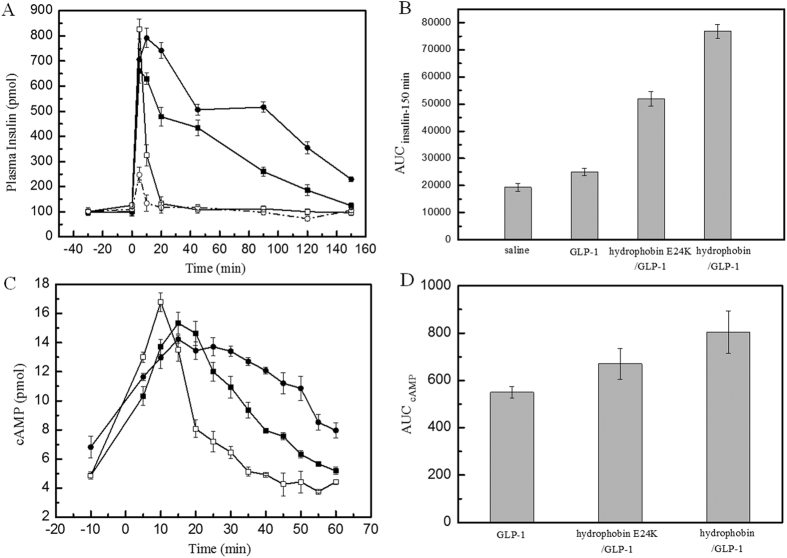
Effects of GLP-1 derivatives on the insulin stimulation and cAMP accumulation after oral glucose administration. Panel A. The stimulation of insulin secretion by native GLP-1 (□) and two complexes (hydrophobin [●] or hydrophobin E24K [■], respectively) in Wistar rats after oral glucose administration. Panel C. The effect of GLP-1 derivatives on cAMP levels in INS-1 cells. LEGEND: The results indicate that the oral administration of glucose increased insulin levels in the rats. The levels of secreted insulin induced by GLP-1 arrived the peak at 10–20 min and returned to baseline at 40 min. The insulin levels in Wistar rats treated with complex showed distinct difference in insulin secretion. Compared with GLP-1, these two complexes stimulated the prolonged secretary response significantly. In similarity, the cellular cAMP level stimulated by GLP-1 peaked to 17.03 ± 0.94 pmol/10^5^ cells in 2 min before declining to baseline at 10 min. However, the cells treated with hydrophobin/GLP-1 (●) or hydrophobin E24K/GLP-1 (■) exhibited longer response than cells treated with GLP-1 remarkably. Conditions: GLP-1 and complexes (100 μg GLP-1/kg body weight) were injected into Wistar rats; glucose was administered orally (10 g/kg). The concentration of insulin was measured using a rat insulin detection kit. Total cellular cAMP was measured in INS-1 cells (1.0 × 10^5^) at the indicated times using an HTRF-cAMP kit. The data are presented as the means ± SE, P < 0.01.

Insulin plays the key role in the glucose regulation induced by GLP-1, indicating that it is necessary to determine whether biological functions such as insulin secretion stimulation and cAMP accumulation were retained following the formation of the complexes. Figure 3A shows that the oral administration of glucose (10 g/kg body weight) increased the insulin levels dramatically to 827.09 ± 37.77 pmol/l at 5 min in the rats treated with GLP-1 only. In rats treated with the hydrophobin/GLP-1 or hydrophobin E24K/GLP-1 complexes, the insulin levels increased to 705.6 ± 43.28 pmol/l and 660.49 ± 47.27 pmol/L, respectively. The rats treated with GLP-1 displayed a shortened insulin secretory response, with their insulin levels returning to baseline 20 min after the glucose infusion. The levels of secreted insulin that were induced by the hydrophobin/GLP-1 or hydrophobin E24K/GLP-1 complexes exhibited remarkably prolonged stimulation duration, with insulin peaking at 10 min and returning to baseline at 120 min. The increased insulin levels, together with the prolonged secretory response of insulin secretion observed in the Wistar rats indicate that the complexes resulted in greater insulin levels over the entire experimental time (150 min) than GLP-1 alone. The AUC_insulin-150 min_ confirmed that the hydrophobin/GLP-1 and hydrophobin E24K/GLP-1 complexes induced 2- and 3-fold increases in insulin levels compared to rats injected with GLP-1, respectively (Fig. 3B). In addition, the cAMP accumulation assay demonstrated increased cAMP levels induced by the hydrophobin/GLP-1 and hydrophobin E24K/GLP-1 complexes (Fig. 3C,D).

### Efficacy period measurement of hydrophobin/GLP-1 and hydrophobin E24K/GLP-1 complexes

**Figure 4:**
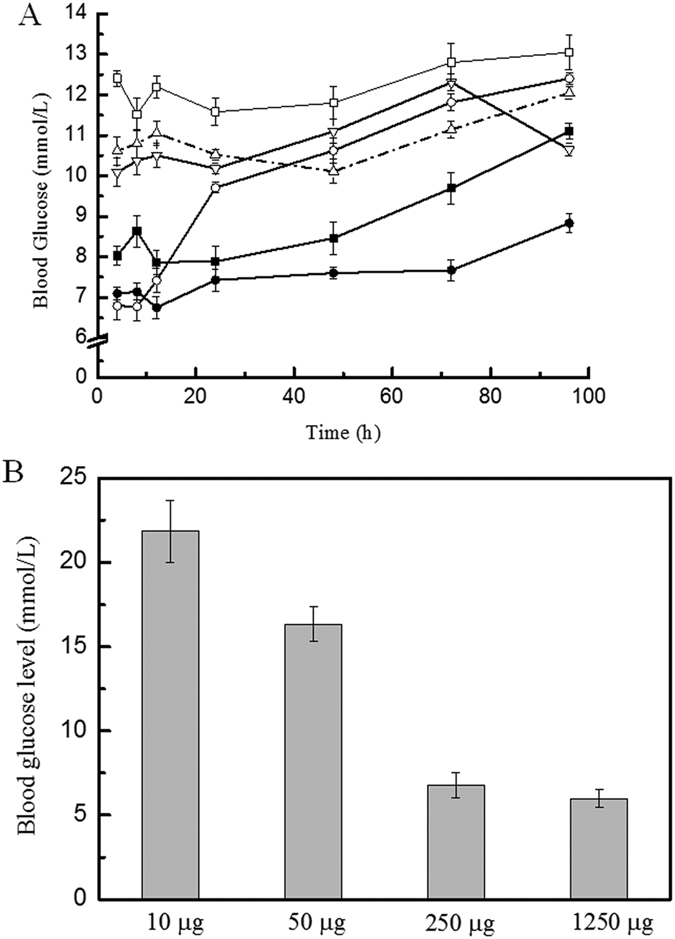
Effects of complexes on glucose tolerance after a single-dose injection. Panel A. The effect of a single injection of the complex and Liraglutide on glucose regulation in Wistar rats. Panel B. The dosage-efficacy of complexes, 48 h after administrations. LEGEND: The rats injected with the hydrophobin/GLP-1 (●) or hydrophobin E24K/GLP-1 complex (■) showed better glucose tolerance than those injected with wild-type GLP-1 (□). The blood glucose levels were maintained at 7–9 mmol/L within 48 h in rats given the hydrophobin E24K/GLP-1 complex (■) and within 72 h in rats given the hydrophobin/GLP-1 complex (●) (Fig. 4A). The pH value appeared to be crucial for the formation of a stable complex; hydrophobin/GLP-1 (pH 4.0) (△) and hydrophobin E24K/GLP-1 (pH 7.0) (▽) have no glucoregulatory effect. Liraglutide loses glucoregulatory activity 24 h after administration (○). Conditions: Fasting Wistar rats were injected with the complex or GLP-1. Glucose (2 g/kg body weight) was administered 30 min before each time point (0–48 h).

The half-lives of the hydrophobin/GLP-1 and hydrophobin E24K/GLP-1 complexes were determined by a single-dose glucose tolerance test in Wistar rats, with liraglutide used as a control. The blood glucose remained at high levels in rats treated with saline or GLP-1 alone. However, the rats injected with the hydrophobin/GLP-1 or hydrophobin E24K/GLP-1 complexes showed better glucose tolerance than those injected with wild-type GLP-1. The blood glucose levels were maintained at 7–9 mmol/L within 48 h in rats given the hydrophobin E24K/GLP-1 complex and within 72 h in rats given the hydrophobin/GLP-1 complex (Fig. 4A). The pH value appeared to be crucial for the formation of a stable complex; hydrophobin/GLP-1 (pH 7.0) and hydrophobin E24K/GLP-1 (pH 3.0) have no glucoregulatory effect. Liraglutide loses glucoregulatory activity 24 h after administration. These results suggest the frequency of hydrophobin/GLP-1 administration could be reduced to one injection every 2–3 days. Furthermore, the results from the dose-efficacy dependent glucoregulatory assay suggested sufficient efficacy at a dose of 250 μg/kg body weight (Fig. 4B).

### Long-term anti-diabetic activity of hydrophobin/GLP-1 and hydrophobin E24K/GLP-1 complexes

**Figure 5:**
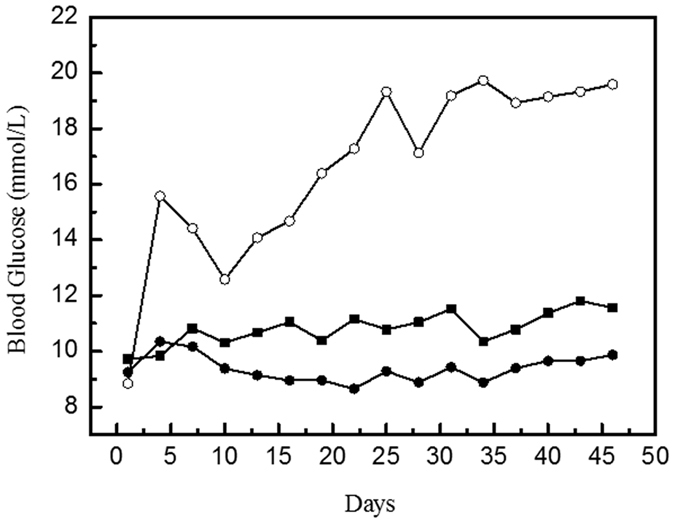
Effect of multiple doses of complexes over 46 days treatment. Panel A. The long-lasting glucose regulatory effect of complexes in ZDF rats. LEGEND: The results indicate that the treated ZDF rats maintained relatively constant and lower glucose levels than the control rats; native GLP-1 failed to produce a similar glucose regulatory effect. Liraglutide is not capable to provide satisfied blood glucose well-control. Conditions: The hydrophobin/GLP-1 (●) and hydrophobin E24K/GLP-1 (■) (250 μg /kg body weight) was administered every three days during the experimental period, which lasted 46 days. A glucometer was used to measure the glucose levels at various time intervals.

To assess if the two complexes possess long-acting anti-diabetic effects, glucose and HbA_1c_ levels were monitored over 46 days. During this experimental period, the hydrophobin/GLP-1 complex, hydrophobin E24K/GLP-1 complex and liraglutide were administered to ZDF rats (250 μg/kg body weight/3 days), and saline was administered to a control group. The blood glucose levels were monitored every 3 days. After 42 days of treatment, the HbA_lc_ levels decreased 1.23 ± 0.06% from 9.23 ± 0.09% (control group), whereas liraglutide only induced a 0.20 ± 0.07% decrease with this dosage frequency. The results clearly indicate that the hydrophobin/GLP-1 complex possessed longer lasting anti-diabetic effects liraglutide (Fig. 5); this result was an improvement compared with the non-clinical data with liraglutide administered at a frequency of one injection every three days^38^.

## Discussion

Many bioactive substances, such as proteins, peptides and nucleic acids, have recently exhibited excellent physiological characteristics and potent clinical utilities. However, their use in clinical practice has been significantly limited due to poor serum stability despite their excellent physiological advantages; an example is glucagon-like peptide-1 (GLP-1)^39^. The known physiological functions of GLP-1 suggest that it plays a critical role in the regulation of glucose homeostasis, and this suggests that it is a feasible candidate in the treatment of type 2 diabetes mellitus^40^,^41^. In addition to its potential role in treatment of T2DM, GLP-1 is also presumed to affect cardiovascular function or cardiovascular disease (CVD) because the GLP-1 receptor has been identified in the heart, kidneys, and blood vessels. The preclinical study of GLP-1 derivatives approved by the FDA might provide evidence that GLP-1 favorably affects endothelial function, sodium excretion, recovery from ischemic injury, and myocardial function in animals. Preliminary data also suggest that GLP-1 reduced markers of CVD risk such as C-reactive protein and plasminogen activator inhibitor-1. Ongoing studies are examining the effects of administering GLP-1 to patients at risk of CVD, postangioplasty patients, post-CABG patients, and patients with heart failure^42^. Despite its attractive physiological characteristics, the therapeutic potential of using native GLP-1 is limited by its short lifetime (<2 min) *in vivo*. This *in vivo* clearance rate is primarily due to rapid enzymatic inactivation by DPP-IV^43^ and a renal clearance of less than 10 min^44^. To provide clinical utility, therapeutic derivatives of human GLP-1 require extended half-life properties. To date, many efforts have focused on altering the pharmacokinetic properties of GLP-1 by developing a series of derivatives and analogs^45^,^46^,^47^, including exenatide, liraglutide and albiglutide and many attempts in laboratories.

**Figure 6:**
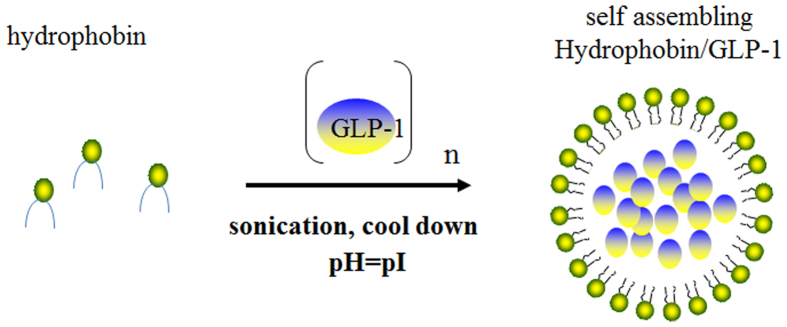
Schematic depicting the formed complex of GLP-1 and hydrophobins.

In this study, we utilized the unique self-assembling property of hydrophobin protein as a stable drug carrier for GLP-1 to achieve long action of glucoregulation. The HPLC assay indicated that the wild-type hydrophobin protein and its mutant E24K are capable of wrapping GLP-1 molecules inside in a self-assembly manner (Fig. 6). However, the different isoelectric points of the wild-type and mutant hydrophobin protein showed unique self-assembling features, as shown in Fig. 1. Furthermore, the wild-type hydrophobin prepared at pH 3.0 and the mutant prepared at pH 7.0 showed self-assembly properties in the HPLC spectra. Two complexes at a ratio of 1:10 were included in the blood retention time measurement. Free GLP-1 released from the complexes remained detectable after 48 and 72 h for wild-type and mutant hydrophobin, respectively. The improved stabilities of the complexes prompted us to examine the prolonged glucoregulatory activity of the hydrophobin/GLP-1 and hydrophobin E24K/GLP-1 complexes. The data demonstrated that treatment with the two complexes resulted in a remarkable increase in insulin (Fig. 3), which suggested longer blood glucose clearance activity than GLP-1 alone. The further single-dose glucose tolerance test validated the prolonged blood glucose clearance activity exhibited by hydrophobin/GLP-1 and hydrophobin E24K/GLP-1. As shown by the data in Fig. 4, the complex of hydrophobin E24K/GLP-1 was still able to control glucose levels at approximately 8–9 mmol/L within 48 h, and the glucose level increased to 9.34 ± 1.56 mmol/L at 72 h after administration. In comparison, the glucose level was well balanced in the rats treated with the complex of wild-type protein and GLP-1 within 72 h. In combination with a dose-dependent assay, the rational dose of the hydrophobin/GLP-1 complex appears to be 250 μg/kg body weight/3 days in further assays. As predicted, the hydrophobin/GLP-1 complex showed remarkably better glucose regulatory activity in the long term than hydrophobin E24K/GLP-1 and liraglutide. The results indicated the hydrophobin/GLP-1 complex is likely a preeminent drug candidate in the treatment of T2DM.

Hydrophobin is a food source protein, suggesting the safety of drug preparation, particularly for chronic diseases including T2DM. The application of a protein as a drug carrier is not unusual for improving drug serum stability; other examples using this technique include human serum albumin (HSA) in abraxane, albiglutide, methotrexate (MTX)-HSA, and albinterferon alpha-2b^48^,^49^,^50^,^51^,^52^,^53^. Most applications of HSA as a drug carrier require chemical conjugation with drugs, which leads to several obstacles in drug release evaluation and efficacy serum stability in the clinic. Abraxane is the first drug approved by the FDA as a complex of HSA and paclitaxel formed by physical interactions. In this study, the self-assembly characteristic of hydrophobin ensures that the drug loading procedure is much easier than abraxane.

The application of hydrophobin protein as a drug carrier might significantly prolong the serum stability of GLP-1. This protein might possess wide possibilities as a drug carrier or in drug preparations, such as with insulin. In addition, by changing the pI through amino acid replacement, the mutated hydrophobin protein might act additionally as a controlled drug release component in the future.

## Additional Information

**How to cite this article**: Zhao, L. *et al.* Novel application of hydrophobin in medical science: a drug carrier for improving serum stability. *Sci. Rep.* **6**, 26461; doi: 10.1038/srep26461 (2016).
